# Prevalence of molecular markers of sulfadoxine-pyrimethamine resistance in *Plasmodium falciparum* isolates from West Africa during 2012–2022

**DOI:** 10.1038/s41598-024-75828-w

**Published:** 2024-11-04

**Authors:** Ruimin Zhou, Suhua Li, Penghui Ji, Shucheng Ruan, Ying Liu, Chengyun Yang, Dan Qian, Zhiquan He, Dan Wang, Deling Lu, Hongwei Zhang, Yan Deng

**Affiliations:** 1 Department of Parasite Disease Control and Prevention, Henan Provincial Key Laboratory of Pathogenic Microbiology, Henan Provincial Medical Key Laboratory of Parasitic Diseases and Vector, Henan Province Center for Disease Control and Prevention, Zhengzhou, People’s Republic of China; 2 College of Public Health, Zhengzhou University, Zhengzhou, People’s Republic of China

**Keywords:** Molecular biology, Diseases, Medical research

## Abstract

Sulfadoxine-pyrimethamine (SP) is a key drug recommended by the World Health Organization for the chemoprevention of malaria. However, the strategy is affected by the parasite resistance to SP. This study evaluated *Plasmodium falciparum* dihydrofolate reductase (*Pfdhfr*) and dihydropteroate synthase (*Pfdhps*) genes, associated with SP resistance, from 508 *P. falciparum* isolates imported from West African countries to Henan Province, China, during 2012–2022. High mutant prevalence of the genes *Pfdhfr* (94.7%) and *Pfdhps* (96.8%) was observed. The mutants *Pfdhfr* N51I, C59R, S108N, and *Pfdhps* A437G were at high frequency in all countries analyzed. The overall prevalence of the mutant *Pfdhps* K540E was low (3.4%), but with a high frequency in Liberia (24.3%). The frequency of mutants *Pfdhps* I431V, A581G, and A613S was 11.7%, 9.8%, and 16.2%, respectively, all of which had the highest mutant prevalence in Nigeria. The mutant *Pfdhps* A581G and A613S were identified in the absence of K540E. The partially resistant haplotype (I_51_R_59_N_108_ - G_437_) was the most common (72.6%), and the fully resistant haplotype (I_51_R_59_N_108_ - G_437_E_540_) had a low prevalence of 3.4% and mainly occurred in Liberia. No super resistant haplotype was identified. The mutant *Pfdhps* I431V and the octuple mutant haplotype I_51_R_59_N_108_ - V_431_A_436_G_437_G_581_S_613_ deserve more attention. In areas of high SP resistance, the intervention still reduces low birthweight and maternal anaemia. SP should continue to be used in areas of high SP resistance until more effective alternatives for malaria chemoprevention are found. It is important to continuously monitor the molecular markers associated with SP resistance to better implement intermittent preventive treatment policies in pregnancy (IPTp) and infants (IPTi).

## Introduction

Malaria is a serious global public health problem, particularly affecting the health of children and pregnant women. Worldwide, there were about 247 million malaria cases in 2021, with estimated 234 million cases in the World Health Organization (WHO) African region, accounting for about 95% of the total^1^. An estimated 13.4 million additional malaria cases were recorded between 2019 and 2021 with disruption to services during the COVID-19 pandemic, and the majority of these also were from countries in the WHO African region^1^. There were 15 malaria-endemic countries in West Africa in 2021, with 120 million estimated malaria cases reported, accounting for 51.3% of the estimated total in the entire WHO African region^1^. Meanwhile, approximately 328,000 deaths occurred in West Africa in 2021, and most deaths (80%) were in children under 5 years of age^1^. In the WHO African Region, about 13.3 million pregnant women were exposed to malaria infection in 2021, with the highest prevalence (40.7%) in West Africa^1^. The pregnant women and children under 5 years old are vulnerable populations, who are at highest risk of severe malaria.

Preventive chemotherapy is an important strategy for malaria control worldwide, especially in areas with moderate to high malaria transmission. Sulfadoxine-pyrimethamine (SP) is recommended by the WHO as a key preventive chemotherapy drug. Due to the spread of chloroquine resistance in the 1960s^2^, SP combination therapy was adopted as a first-line drug to treat uncomplicated *Plasmodium falciparum*. It too was soon withdrawn as a treatment for clinical malaria because of resistance emergence^3^. However, it continued to be recommended for intermittent preventive treatment of malaria in pregnancy (IPTp) and infants (IPTi)^4–6^. The drug has been shown to be safe, effective, and cost-effective in reducing disease burden and saving lives. Recently updated and published WHO guidance^7^ includes three key strategies: seasonal malaria chemoprevention (SMC), perennial malaria chemoprevention (PMC) as a replacement for IPTi, and intermittent preventive treatment of malaria in pregnancy (IPTp). The strong recommendation by WHO has reaffirmed the use of IPTp-SP in areas with moderate to high malaria transmission. Compared with the original recommendations, the updated guidelines are less restrictive regarding age, dose, transmission intensity, and specific drugs.

Nevertheless, the preventive effectiveness of SP is affected by the parasite resistance to the drugs. *P. falciparum* resistance to SP is affected by single nucleotide polymorphisms at specific codons in the two genes *P. falciparum* dihydropteroate synthase (*Pfdhps*) and dihydrofolate reductase (*Pfdhfr*), both of which are involved in the folate biosynthesis pathway. The polymorphisms of the *Pfdhps* and *Pfdhfr* genes are associated with resistance to sulfadoxine and pyrimethamine, respectively^8,9^. Most studies have indicated that SP resistance is related to mutations at codons S436A/F, A437G, K540E, A581G, and A613S/T in *Pfdhps* genes and N51I, C59R, S108N, and I164L in *Pfdhfr* genes^10–13^. A novel mutation I431V in *Pfdhps* genes was first detected in Nigeria in 2007, then in some other countries of West and Central Africa^14–17^. In most cases, the mutation I431V occurs simultaneously with four other *Pfdhps* mutations: S436A, A437G, A581G, and A613S, forming a quintuple mutant **V**_431_**A**_436_**G**_437_K_540_**G**_581_**S**_613_^18^. The combination of mutations in *Pfdhfr* and *Pfdhps* genes is strongly correlated with the clinical failure of SP treatment^19–21^. In addition, the type and number of mutant codons affect the level of SP resistance. Resistance levels are classified as follows: partially resistant consists of mutations of *Pfdhfr* I_51_R_59_N_108_ and *Pfdhps* G_437_ (I_51_R_59_N_108_-G_437_); fully resistant additionally includes *Pfdhfr* I_51_R_59_N_108_ and *Pfdhps* G_437_E_540_ (I_51_R_59_N_108_-G_437_E_540_); and the super resistant classification includes fully resistant mutations and the additional mutants *Pfdhfr* I164L and/or *Pfdhps* A581G and/or A613S/T^22^.

It is essential to monitor the effectiveness of drugs for the better implementation of preventive chemotherapy strategies in malaria control. Surveillance of the molecular markers associated with drug resistance is one way to assess its spread. The *Pfdhps* and *Pfdhfr* genes associated with SP resistance are used to evaluate the effectiveness of SP in the SMC, PMC, and IPTp chemoprevention strategies. This study aimed to assess the distribution and prevalence of *Pfdhps* and *Pfdhfr* genes in imported cases of *P. falciparum* from West African countries to Henan Province during 2012–2022 and to add to the knowledge on molecular markers related to SP resistance.

## Results

### General characteristics

A total of 508 *P. falciparum* infections imported from West Africa were reported in Henan Province, China, during 2012 − 2022. All 508 cases were imported from 11 countries in West Africa, with six countries accounting for more than 90% of the cases: Nigeria (35.0%, 178/508), Guinea (16.3%, 83/508), Ghana (12.4%, 63/508), Sierra Leone (10.0%, 51/508), Côte d’Ivoire (9.3%, 47/508), and Liberia (8.7%, 44/508) (Table 1). The temporal distribution of cases was relatively stable from 2012 to 2019; however, the number of cases decreased sharply due to the COVID-19 pandemic in 2020 − 2022 (Table 1). All but one Nigerian and one Liberian were Chinese citizens who traveled to countries in West Africa and returned with malaria infection. The ratio of female to male was 1:38.1, with a total of 13 females. The average age was 38.36 ± 10.16 years (range, 19 − 70 years). Among all cases, migrant workers accounted for 77.4% (393/508).

**Table 1:** *Plasmodium falciparum* infected cases imported from west African countries to Henan Province, China, during 2012–2022.

Countries	2012	2013	2014	2015	2016	2017	2018	2019	2020	2021	2022	Total (%)
Nigeria	23	14	28	13	18	27	15	31	4	1	4	178 (35.0)
Guinea	8	9	4	17	4	7	12	19	1	0	2	83 (16.3)
Ghana	5	8	6	8	13	8	6	7	1	0	1	63 (12.4)
Sierra Leone	10	7	8	6	7	4	3	4	0	1	1	51 (10.0)
Côte d’Ivoire	1	0	3	5	3	9	7	16	3	0	0	47 (9.3)
Liberia	5	10	8	5	2	8	5	1	0	0	0	44 (8.7)
Benin	0	2	5	0	1	1	0	10	0	0	0	19 (3.7)
Togo	0	0	6	0	0	0	2	1	1	0	0	10 (2.0)
Mali	1	1	3	0	0	0	0	2	0	0	0	7 (1.4)
Senegal	0	0	0	0	2	0	1	1	0	0	1	5 (1.0)
Burkina Faso	0	0	0	1	0	0	0	0	0	0	0	1 (0.2)
Total (%)	53 (10.4)	51 (10.0)	71 (14.0)	55 (10.8)	50 (9.8)	64 (12.6)	51 (10.0)	92 (18.1)	10 (2.0)	2 (0.4)	9 (1.8)	508 (100.0)

### *Pfdhfr* alleles

**Fig. 1:**
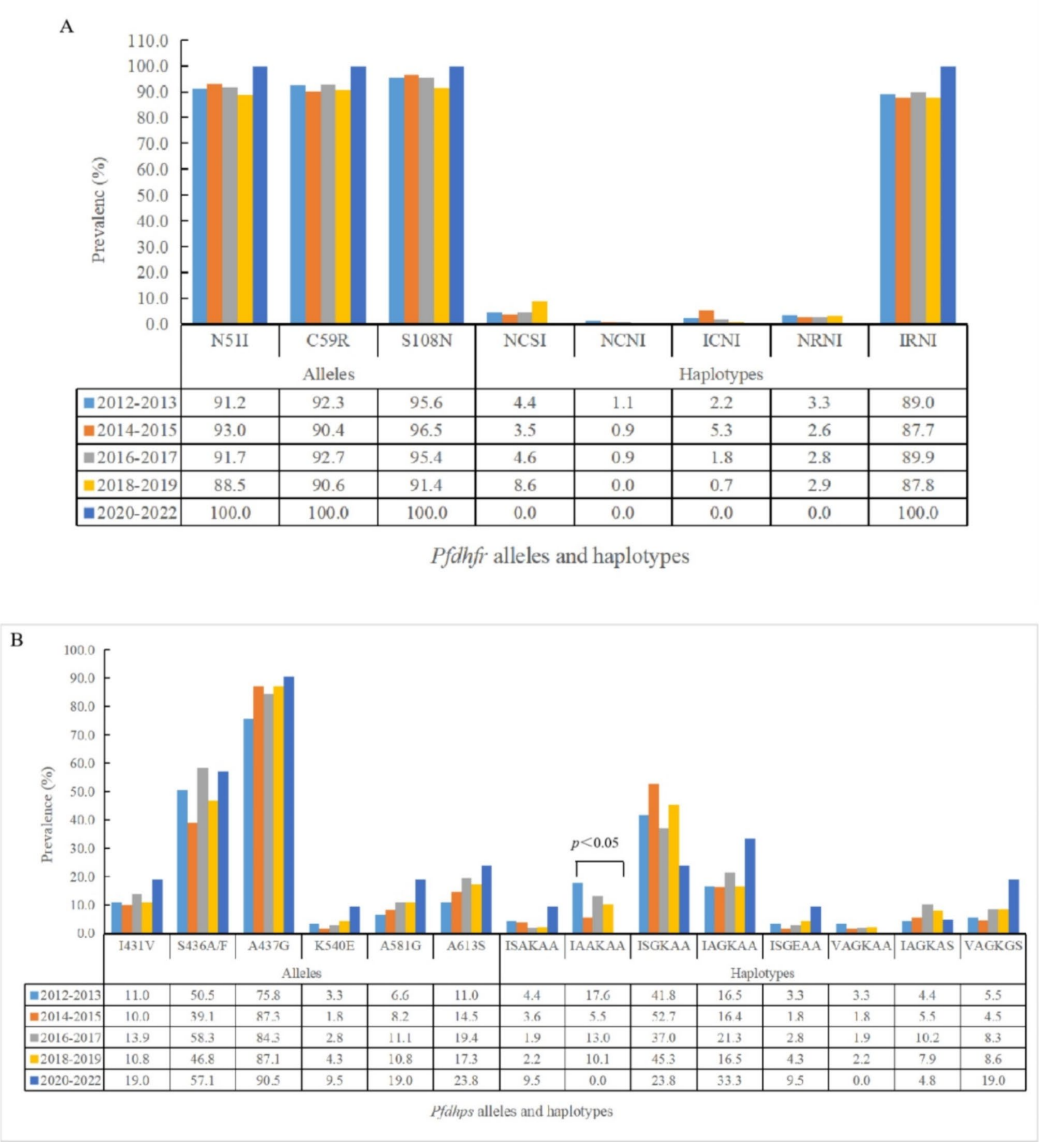
Prevalence of alleles and haplotypes in *Pfdhfr* (A) and *Pfdhps* (B) genes detected in *Plasmodium falciparum* isolates from West African countries during 2012–2022.

The *Pfdhfr* gene was successfully sequenced from 473 *P. falciparum* isolates. Six mutant alleles of the *Pfdhfr* gene were identified: N51I, C59R, D87H, S108N, S120R, and I164L. The mutant alleles D87H, S120R, and I164L were detected in only one isolate each. The mutant D87H allele was isolated from the sample from Guinea, and the mutant S120R and I164L alleles were identified from Nigeria. By contrast, the prevalence of the mutant N51I, C59R, and S108N alleles was high and appeared in all 11 countries sampled, accounting for 91.3% (432/473), 91.8% (434/473), and 94.7% (448/473), respectively (Table 2). Compared with other countries, the prevalence of the mutant S108N alleles was lower in Senegal, Côte d’Ivoire, Togo, and Guinea (*x*^2^ = 17.434, *p* < 0.05). There was no significant difference in the prevalence of the mutant N51I, C59R, and S108N alleles from 2012 to 2022 (Fig. 1A).

**Table 2:** Prevalence of *pfdhfr* alleles and haplotypes detected in *Plasmodium falciparum* isolates from west African countries.

Country (No. of isolates)	Alleles*			Haplotypes^#^				
	N51I	C59R	S108N	NCSI	NCNI	ICNI	NRNI	IRNI
Total (*n* = 473)	432 (91.3)	434 (91.8)	448 (94.7)	25 (5.3)	3 (0.6)	11 (2.3)	13 (2.7)	420 (88.8)
Nigeria (*n* = 171)	159 (93.0)	158 (92.4)	166 (97.1)	5 (2.9)	2 (1.2)	6 (3.5)	5 (2.9)	152 (88.9)
Guinea (*n* = 79)	70 (88.6)	69 (87.3)	71 (89.9)	8 (10.1)	1 (1.3)	1 (1.3)	0	69 (87.3)
Ghana (*n* = 59)	53 (89.8)	56 (94.9)	57 (96.6)	2 (3.4)	0	1 (1.7)	4 (6.8)	52 (88.1)
Côte d’Ivoire (*n* = 45)	36 (80.0)	39 (86.7)	39 (86.7)	6 (13.3)	0	0	3 (6.7)	36 (80.0)
Sierra Leone (*n* = 44)	41 (93.2)	41 (93.2)	42 (95.5)	2 (4.5)	0	1 (2.3)	1 (2.3)	40 (90.9)
Liberia (*n* = 37)	37 (100)	36 (97.3)	37 (100)	0	0	1 (2.7)	0	36 (97.3)
Benin (*n* = 17)	17 (100)	17 (100)	17 (100)	0	0	0	0	17 (100)
Togo (*n* = 9)	8 (88.9)	8 (88.9)	8 (88.9)	1 (11.1)	0	0	0	8 (88.9)
Mali (*n* = 6)	6 (100)	5 (83.3)	6 (100)	0.0	0	1 (16.7)	0	5 (83.3)
Senegal (*n* = 5)	4 (80.0)	4 (80.0)	4 (80.0)	1 (20.0)	0	0	0	4 (80.0)
Burkina Faso (*n* = 1)	1 (100)	1 (100)	1 (100)	0	0	0	0	1 (100)
*x* ^2^	15.543	10.657	17.434	17.434	10.678	9.035	11.009	10.606
*P* value	0.079	0.324	0.039	0.039	1.000	0.567	0.326	0.336

*The mutant D87H allele was isolated from the sample of Guinea; the mutant S120R and I164L alleles were all identified from Nigeria.

^#^One haplotype IRNL was detected in the isolate from Nigeria in 2012.

Boldface type indicates the mutant amino acid.

### *Pfdhfr* haplotypes

The mutant D87H and S120R alleles of the *Pfdhfr* gene were excluded from the haplotype construction. Using the codons N51I, C59R, S108N, and I164L in the *Pfdhfr* gene, six haplotypes were observed among the 473 isolates. The wild-type haplotype N_51_C_59_S_108_I_164_ was carried by 5.3% (25/473) of the isolates. The prevalence of wild-type haplotype NCSI from Senegal, Côte d’Ivoire, and Guinea was lower than from other countries (*x*^2^ = 17.434, *p* < 0.05). The most common mutant haplotype of the *Pfdhps* gene was the triple mutant **IRN**I (88.8%, 420/473), followed by the double mutants N**RN**I (2.7%, 13/473) and **I**C**N**I (2.3%, 11/473). The single mutant NC**N**I was identified in three isolates, and the quadruple mutant **IRNL** was detected in only one isolate from Nigeria (Table 2). There was no significant difference in the prevalence of the haplotypes NCSI, NC**N**I, **I**C**N**I, N**RN**I, and **IRN**I from 2012 to 2022 (Fig. 1A).

### *Pfdhps* alleles

For the *Pfdhps* gene, 469 isolates were successfully sequenced. Nine mutant alleles were identified: I431V, S436A, S436F, A437G, I484T, K540E, A581G, A613S, and H614L. The mutant A437G allele was the most common and identified in all 11 countries, accounting for 84.4% (396/469). However, the prevalence of mutant A437G in Senegal (40.0%, 2/5), Mali (66.7%, 4/6), Guinea (72.4%, 56/77), and Sierra Leone (74.4%, 32/43) was lower than in other countries (*x*^2^ = 29.067, *p* < 0.01). There were two types of mutations at codon 436: S436A (47.8%, 224/469) and S436F (1.1%, 5/469). Almost half of the *Pfdhps* isolates (48.8%, 229/469) harbored the mutant S436A/F alleles. The mutant S436F allele was identified in four isolates from Nigeria and one from Benin and was always coexistent with the A613S allele. The mutant A613S, I431V, A581G, and K540E alleles were detected in 16.2% (76/469), 11.7% (55/469), 9.8% (46/469), and 3.4% (16/469) of the isolates, respectively (Table 3). The mutant I484T and H614L alleles were rarely reported, each of them was detected in two isolates in this study. Prevalences of the mutant I431V (27.5%, 47/171), A581G (22.2%, 38/171), and A613S (29.2%, 50/171) alleles in Nigeria were all significantly higher than in other countries (I431V, *x*^2^ = 73.540, *p <* 0.01; A581G, *x*^2^ = 55.398, *p <* 0.01; A613S, *x*^2^ = 43.284, *p <* 0.01). Meanwhile, the proportion of the K540E allele in Liberia (24.3%, 9/37) was significantly higher than that in other countries (*x*^2^ = 39.064, *p <* 0.01). As shown in Fig. 1B, the frequency of the I431V, S436A/F, A437G, K540E, A581G, and A613S alleles all showed a trend of increasing from 2012 to 2022, but the difference was not statistically significant.

**Table 3:** Prevalence of *Pfdhps* alleles and haplotypes detected in *Plasmodium falciparum* isolates from west African countries.

Alleles/haplotypes	Total (*n* = 469)	Nigeria (*n* = 171)	Guinea (*n* = 77)	Ghana (*n* = 58)	Côte d’Ivoire (*n* = 45)	Sierra Leone (*n* = 43)	Liberia (*n* = 37)	Benin (*n* = 17)	Togo (*n* = 9)	Mali (*n* = 6)	Senegal (*n* = 5)	Burkina Faso (*n* = 1)	x^2^	*P* value
Alleles														
I431V	55 (11.7)	47 (27.5)	0	1 (1.7)	2 (4.4)	1 (2.3)	3 (8.1)	1 (5.9)	0	0	0	0	73.540	0.000
S436A/F	229 (48.8)	88 (51.5)	31 (40.3)	35 (60.3)	24 (53.3)	20 (46.5)	13 (35.1)	7 (41.2)	4 (44.4)	5 (83.3)	2 (40.0)	0	14.202	0.164
A437G	396 (84.4)	148 (86.5)	56 (72.7)	55 (94.8)	40 (88.9)	32 (74.4)	33 (89.2)	16 (94.1)	9 (100)	4 (66.7)	2 (40.0)	1 (100)	29.067	0.001
K540E	16 (3.4)	0	1 (1.3)	2 (3.4)	1 (2.2)	3 (7.0)	9 (24.3)	0	0	0	0	0	39.064	0.000
A581G	46 (9.8)	38 (22.2)	0	1 (1.7)	2 (4.4)	1 (2.3)	3 (8.1)	1 (5.9)	0	0	0	0	55.398	0.000
A613S	76 (16.2)	50 (29.2)	5 (6.5)	2 (3.4)	7 (15.6)	3 (7.0)	5 (13.5)	3 (17.6)	0	1 (16.7)	0	0	43.284	0.000
Wild-type														
I_431_S_436_A_437_K_540_A_581_A_613_	15 (3.2)	6 (3.5)	4 (5.2)	0	1 (2.2)	2 (4.7)	1 (2.7)	0	0	0	1 (20.0)	0	9.390	0.525
Single mutant														
IS**G**KAA	204 (43.5)	72 (42.1)	41 (53.2)	21 (36.2)	19 (42.2)	18 (41.9)	14 (37.8)	10 (58.8)	5 (55.6)	1 (16.7)	2 (40.0)	1 (100)	10.713	0.380
I**A**AKAA	50 (10.7)	11 (6.4)	17 (22.1)	3 (5.2)	4 (8.9)	9 (20.9)	2 (5.2)	0	0	2 (33.3)	2 (40.0)	0	30.733	0.001
Double mutant														
I**AG**KAA	86 (18.3)	15 (8.8)	9 (11.7)	28 (48.3)	13 (28.9)	7 (16.3)	4 (10.8)	4 (23.5)	4 (44.4)	2 (33.3)	0	0	53.179	0.000
IS**GE**AA	16 (3.4)	0	1 (1.3)	2 (3.4)	1 (2.2)	3 (7.0)	9 (24.3)	0	0	0	0	0	39.064	0.000
I**F**AKA**S**	5 (1.1)	4 (2.3)	0	0	0	0	0	1 (5.9)	0	0	0	0	9.803	0.458
IS**G**K**G**A	3 (0.6)	3 (1.8)	0	0	0	0	0	0	0	0	0	0	6.087	0.808
**VA**AKAA	2 (0.4)	2 (1.2)	0	0	0	0	0	0	0	0	0	0	4.051	0.945
Triple mutant														
I**AG**KA**S**	33 (7.0)	13 (7.6)	5 (6.5)	2 (3.4)	5 (11.1)	3 (7.0)	3 (8.1)	1 (5.9)	0	1 (16.7)	0	0	5.408	0.862
**VAG**KAA	10 (2.1)	8 (4.7)	0	1 (1.7)	0	0	1 (2.7)	0	0	0	0	0	12.832	0.233
**VA**AK**G**A	1 (0.2)	0	0	0	0	0	1 (2.7)	0	0	0	0	0	5.105	0.884
**V**S**G**K**G**A	1 (0.2)	1 (0.6)	0	0	0	0	0	0	0	0	0	0	2.022	0.996
I**AG**K**G**A	1 (0.2)	0	0	1 (1.7)	0	0	0	0	0	0	0	0	4.196	0.938
Quadruple mutant														
**VAG**K**G**A	3 (0.6)	3 (1.8)	0	0	0	0	0	0	0	0	0	0	6.087	0.808
**VAG**KA**S**	2 (0.4)	2 (1.2)	0	0	0	0	0	0	0	0	0	0	4.051	0.945
**V**S**G**K**GS**	1 (0.2)	1 (0.6)	0	0	0	0	0	0	0	0	0	0	2.022	0.996
I**AG**K**GS**	1 (0.2)	0	0	0	0	0	1 (2.7)	0	0	0	0	0	5.105	0.884
Quintuple mutant														
**VAG**K**GS**	35 (7.5)	30 (17.5)	0	0	2 (4.4)	1 (2.3)	1 (2.7)	1 (5.9)	0	0	0	0	47.498	0.000

Boldface type indicates the mutant amino acid.

### *Pfdhps* haplotypes

The mutant I484T and H614L alleles were excluded to construct the haplotypes of the *Pfdhfr* gene. This study identified 18 *Pfdhps* haplotypes, including wild-type, single, double, triple, quadruple, and quintuple mutations. Only 15 isolates were without any point mutant, accounting for 3.2% (15/469). Single-mutant haplotypes were common, including IS**G**KAA (43.5%, 204/469) and I**A**AKAA (10.7%, 50/469). The I**A**AKAA haplotype proportions in Senegal (40.0%, 2/5), Guinea (22.1%, 17/77), and Sierra Leone (20.9%, 9/43) were significantly higher than in other countries (*x*^2^ = 30.733, *p* < 0.01). Five double mutant haplotypes were detected, including I**AG**KAA (18.3%, 86/469), IS**GE**AA (3.4%, 16/469), I**F**AKA**S** (1.1%, 5/469), IS**G**K**G**A (0.6%, 3/469), and **VA**AKAA (0.4%, 2/469). The prevalence of the I**AG**KAA haplotype in Ghana (48.3%, 28/58), Togo (44.4%, 4/9), and Mali (33.3%, 2/6) was significantly higher than in other countries (*x*^2^ = 53.179, *p <* 0.01). The highest prevalence of the IS**GE**AA haplotype was identified in Liberia, at 24.3% (9/37) among the countries in the study (*x*^2^ = 39.064, *p <* 0.01). Of the five triple mutant haplotypes detected, the **VA**AK**G**A, **V**S**G**K**G**A, and I**AG**K**G**A haplotypes were identified in one isolate. The other two were I**AG**KA**S** (7.0%, 33/469) and **VAG**KAA (2.1%, 10/469). Similarly, four quadruple haplotypes were detected with low prevalence: **VAG**K**G**A (0.6%, 3/469), **VAG**KA**S** (0.4%, 2/469), **V**S**G**K**GS** (0.2%, 1/469), and I**AG**K**GS** (0.2%, 1/469). The quintuple mutant haplotype **VAG**K**GS** was found in isolates from five countries: Nigeria (17.5%, 30/469), Côte d’Ivoire (4.4%, 2/45), Sierra Leone (2.3%, 1/43), Liberia (2.7%, 1/37), and Benin (5.9%, 1/17), with Nigeria having the highest prevalence (*x*^2^ = 47.498, *p <* 0.01) (Table 3). The prevalence of I**A**AKAA haplotype decreased from 2012 to 2022 (*x*^2^ = 10.865, *p* < 0.05), while there was no significant change in that of the other haplotypes (Fig. 1B).

### Combination of the *Pfdhfr* and *Pfdhps* Haplotypes

Among all 508 *P. falciparum* cases, 468 isolates were successfully sequenced for the *Pfdhfr* and *Pfdhps* genes. A total of 37 haplotypes were obtained by combining the *Pfdhfr* and *Pfdhps* haplotypes. Five isolates were wild type, accounting for 1.1% (5/468). The **IRN**I-IS**G**KAA haplotype (39.1%, 183/468) was most prevalent in West Africa, followed by **IRN**I-I**AG**KAA (16.0%, 75/468), **IRN**I-I**A**AKAA (9.6%, 45/468), **IRN**I-**VAG**K**GS** (7.3%, 34/468), and **IRN**I-I**AG**KA**S** (6.0%, 28/468). Table 4 shows detailed information. The partially resistant haplotype with the **I**_51_**R**_59_**N**_108_ - **G**_437_ mutant was identified in 72.6% (340/468) of the isolates, including 13 haplotypes of the combined *Pfdhfr* and *Pfdhps* gene. There was no significant difference in the prevalence of the partially resistant haplotype among the countries (*x*^2^ = 17.332, *p* > 0.05). The fully resistant haplotype with the **I**_51_**R**_59_**N**_108_ - **G**_437_**E**_540_ mutant accounted for 3.4% (16/468) and was identified in five countries: Guinea (1.3%, 1/77), Ghana (3.4%, 2/58), Côte d’Ivoire (2.3%, 1/44), Sierra Leone (7.0%, 3/43), and Liberia (24.3%, 9/37). Liberia had the highest prevalence of the fully resistant haplotype compared with other countries (*x*^2^ = 37.875, *p <* 0.01). No super resistant haplotype was found in our study.

**Table 4:** Prevalence of *Pfdhfr*-*Pfdhps* haplotypes detected in *Plasmodium falciparum* isolates from west African countries.

Pfdhfr and Pfdhps haplotypes	No. of mutant codons	Nigeria (*n* = 171)	Guinea (*n* = 77)	Ghana (*n* = 58)	Côte d’Ivoire (*n* = 44)	Sierra Leone (*n* = 43)	Liberia (*n* = 37)	Benin (*n* = 17)	Togo (*n* = 9)	Mali (*n* = 6)	Senegal (*n* = 5)	Burkina Faso (*n* = 1)	Total (*n* = 468)
Undefinitive resistant													
NCSI-ISAKAA	0	1 (0.6)	2 (2.6)	0	1 (2.3)	1 (2.3)	0	0	0	0	0	0	5 (1.1)
NCSI-IS**G**KAA	1	2 (1.2)	3 (3.9)	2 (3.4)	1 (2.3)	0	0	0	1 (11.1)	0	0	0	9 (1.9)
NCSI-I**A**AKAA	1	0	1 (1.3)	0	0	1 (2.3)	0	0	0	0	1 (20.0)	0	3 (0.6)
NC**N**I-ISAKAA	1	0	1 (1.3)	0	0	0	0	0	0	0	0	0	1 (0.2)
NCSI-I**AG**KAA	2	0	1 (1.3)	0	2 (4.5)	0	0	0	0	0	0	0	3 (0.6)
NC**N**I-IS**G**KAA	2	1 (0.6)	0	0	0	0	0	0	0	0	0	0	1 (0.2)
**I**C**N**I-ISAKAA	2	0	0	0	0	0	1 (2.7)	0	0	0	0	0	1 (0.2)
NCSI-I**AG**KA**S**	3	1 (0.6)	0	0	2 (4.5)	0	0	0	0	0	0	0	3 (0.6)
NCSI-**VAG**KAA	3	1 (0.6)	0	0	0	0	0	0	0	0	0	0	1 (0.2)
**I**C**N**I-IS**G**KAA	3	2 (1.2)	1 (1.3)	0	0	0	0	0	0	0	0	0	3 (0.6)
**I**C**N**I-I**A**AKAA	3	0	0	0	0	1 (2.3)	0	0	0	1 (16.7)	0	0	2 (0.4)
**IRN**I-ISAKAA	3	5 (2.9)	1 (1.3)	0	0	1 (2.3)	0	0	0	0	1 (20.0)	0	8 (1.7)
N**RN**I-IS**G**KAA	3	2 (1.2)	0	3 (5.2)	2 (4.5)	0	0	0	0	0	0	0	7 (1.5)
NC**N**I-I**AG**KA**S**	4	1 (0.6)	0	0	0	0	0	0	0	0	0	0	1 (0.2)
**IRN**I-I**A**AKAA	4	11 (6.4)	16 (20.8)	3 (5.2)	4 (9.1)	7 (16.3)	2 (5.4)	0	0	1 (16.7)	1 (20.0)	0	45 (9.6)
**I**C**N**I-I**AG**KAA	4	3 (1.8)	0	1 (1.7)	0	0	0	0	0	0	0	0	2 (0.4)
**I**C**N**I-IS**G**K**G**A	4	1 (0.6)	0	0	0	0	0	0	0	0	0	0	1 (0.2)
N**RN**I-I**AG**KAA	4	1 (0.6)	0	1 (1.7)	1 (2.3)	1 (2.3)	0	0	0	0	0	0	2 (0.4)
N**RN**I-**VAG**KAA	5	1 (0.6)	0	0	0	0	0	0	0	0	0	0	1 (0.2)
N**RN**I-I**AG**KA**S**	5	1 (0.6)	0	0	0	0	0	0	0	0	0	0	1 (0.2)
**IRN**I-I**F**AKA**S**	5	4 (2.3)	0	0	0	0	0	1 (5.9)	0	0	0	0	5 (1.1)
**IRN**I-**VA**AKAA	5	2 (1.2)	0	0	0	0	0	0	0	0	0	0	2 (0.4)
**IRN**I-**VA**AK**G**A	6	0	0	0	0	0	1 (2.7)	0	0	0	0	0	1 (0.2)
Subtotal		40 (23.4)	26 (33.8)	10 (17.2)	13 (29.5)	12 (27.9)	4 (10.8)	1 (5.9)	1 (11.1)	2 (33.3)	3 (60.0)	0	112 (23.9)
Partially resistant													
**IRN**I-IS**G**KAA	4	64 (37.4)	37 (48.1)	16 (27.6)	16 (36.4)	18 (41.9)	14 (37.8)	10 (58.8)	4 (44.4)	1 (16.7)	2 (40.0)	1 (100)	183 (39.1)
**IRN**I-I**AG**KAA	5	11 (6.4)	8 (10.4)	26 (44.8)	10 (22.7)	6 (14.0)	4 (10.8)	4 (23.5)	4 (44.4)	2 (33.3)	0	0	75 (16.0)
**IRN**I-IS**G**K**G**A	5	2 (1.2)	0	0	0	0	0	0	0	0	0	0	2 (0.4)
**IRNL-**IS**G**KAA	5	1 (0.6)	0	0	0	0	0	0	0	0	0	0	1 (0.2)
**IRN**I-**V**S**G**K**G**A	6	1 (0.6)	0	0	0	0	0	0	0	0	0	0	1 (0.2)
**IRN**I**-**I**AG**KA**S**	6	10 (5.8)	5 (6.5)	2 (3.4)	3 (6.8)	3 (7.0)	3 (8.1)	1 (5.9)	0	1 (16.7)	0	0	28 (6.0)
**IRN**I-**VAG**KAA	6	6 (3.5)	0	1 (1.7)	0	0	1 (2.7)	0	0	0	0	0	8 (1.7)
**IRN**I-I**AG**K**G**A	6	0	0	1 (1.7)	0	0	0	0	0	0	0	0	1 (0.2)
**IRN**I-**VAG**K**G**A	7	3 (1.8)	0	0	0	0	0	0	0	0	0	0	3 (0.6)
**IRN**I-**VAG**KA**S**	7	2 (1.2)	0	0	0	0	0	0	0	0	0	0	2 (0.4)
**IRN**I-**V**S**G**K**GS**	7	1 (0.6)	0	0	0	0	0	0	0	0	0	0	1 (0.2)
**IRN**I-I**AG**K**GS**	7	0	0	0	0	0	1 (2.7)	0	0	0	0	0	1 (0.2)
**IRN**I-**VAG**K**GS**	8	30 (17.5)	0	0	1 (2.3)	1 (2.3)	1 (2.7)	1 (5.9)	0	0	0	0	34 (7.3)
Subtotal		131 (76.6)	50 (64.9)	46 (79.3)	30 (68.2)	28 (65.1)	24 (64.9)	16 (94.1)	8 (88.9)	4 (66.7)	2 (40.0)	1 (100)	340 (72.6)
Fully resistant													
**IRN**I-IS**GE**AA	5	0	1 (1.3)	2 (3.4)	1 (2.3)	3 (7.0)	9 (24.3)	0	0	0	0	0	16 (3.4)

Boldface type indicates the mutant amino acid.

## Discussion

Malaria in West Africa accounted for almost half of all cases globally in 2021, with Nigeria having the highest prevalence worldwide^1^. In this study, 508 *P. falciparum*-infected cases were imported from 11 out of the 15 malaria-endemic countries in West Africa, with Nigeria (35.0%) and Guinea (16.3%) accounting for half of the total cases. The recommended strategy of using SP to treat pregnant women with malaria, known as IPTp-SP, has played an important role in reducing neonatal mortality, low birthweight, and maternal anemia^22,23^. However, the effectiveness of IPTp-SP is threatened by the high prevalence and spread of SP resistance, especially in Africa^24^.

Mutations in the *Pfdhfr* gene have been attributed to the inhibition constant (*Ki*) for pyrimethamine, and the *Pfdhfr* mutations N51I, C59R, S108N, and I164L are considered critical for the generation of pyrimethamine resistance^25^. The *Pfdhfr* mutant alleles N51I, C59R, and S108N have been found to be highly prevalent and widespread in Africa, as well as the triple mutant *Pfdhfr* I_51_R_59_N_108_ haplotype. Almost all had a prevalence of over 90%^17,23–26^. Previous research also showed the mutant *Pfdhfr* I164L had a high frequency in Asia, with relatively low prevalence in Africa^27–29^. Similarly, our study revealed that the *Pfdhfr* mutants N51I, C59R, and S108N had more than 90% prevalence in West African countries, except for Senegal, Côte d’Ivoire, Togo, and Guinea, which had less. The triple mutant **IRN**I (88.8%) was the most common haplotype of *Pfdhfr.* Meanwhile, the mutation I164L was detected in only one isolate from Nigeria in 2012. The prevalence of the mutants N51I, C59R, and S108N did not change significantly during the years 2012–2022. This was probably because those mutants had reached a very high frequency, and the mutations were fixed in the parasite population. It has been inferred that S108N, as a critical intermediate mutant, occurred first. Then, as it was followed by either N51I, C59R, and/or I164L mutations, the level of pyrimethamine resistance increased further^25^. As shown in Table 2, the *Pfdhfr* S108N mutant in our study had higher or equivalent frequency compared with N51I and C59R in all countries.

For the *Pfdhps* gene, the mutant alleles A437G and K540E are key to SP resistance. Studies have shown that the triple mutant haplotype I_51_R_59_N_108_, in combination with the mutant *Pfdhps* A437G and K540E to form the quintuple mutant haplotype I_51_R_59_N_108_ - G_437_E_540_, is strongly associated with an increased risk of SP treatment failure^30,31^. The prevalence of the triple mutant haplotype I_51_R_59_N_108_ and the mutant *Pfdhps* A437G is very high in Africa, so the frequency of the mutant *Pfdhps* K540E could be used as a surrogate marker for the quintuple haplotype I_51_R_59_N_108_ - G_437_E_540_. The sextuple haplotype I_51_R_59_N_108_ - G_437_E_540_G_518_ is formed with the additional mutation of *Pfdhps* A581G and has been associated with the increase of the IPTp-SP, resulting in low infant birth weight and increasing placental inflammation^32–34^.

In 2010, the IPTi-SP was not recommended for areas where the prevalence of the mutant *Pfdhps* K540E exceeded 50%; however, the presence of the mutant *Pfdhps* A581G did not yet influence IPTi policy^35^. In 2013, the WHO Evidence Review Group recommended that IPTp-SP should be discontinued when the prevalence of the mutant *Pfdhps* K540E exceeded 95% and the prevalence of the mutant *Pfdhps* A581G exceeded 10%, as SP was likely to be ineffective^36^. In the current WHO guidelines, the prevalence of mutations associated with SP resistance is no longer a prerequisite for the use of IPTp-SP^7^. A study of SP resistance across Africa from 1990 to 2020 showed that the mutant *Pfdhps* A437G was very common across Africa, the mutant *Pfdhps* K540E was highly prevalent in East and Southeast Africa and relatively low in other regions of Africa, and the mutant *Pfdhps* A581G was identified across Africa, but its prevalence was relatively low^37^. Similarly, our study showed the frequency of the mutant *Pfdhps* A437G, K540E, and A581G alleles at 84.4%, 3.4%, and 9.8%, respectively. The prevalence of K540E was relatively low and was mainly identified in Liberia. The prevalence of A581G was close to 10% and was mainly detected in Nigeria. It was previously reported that mutants *Pfdhps* A581G and A613S were detected in the absence of K540E in West Africa^24^, which our study confirmed. Furthermore, K540E was always detected as coexistent with A437G.

The mutant *Pfdhps* I431V was first identified in Nigeria, then its prevalence increased and was subsequently reported in countries of West and Central Africa^38,39^. Our study found the I431V mutation in samples from six countries, with the highest prevalence in Nigeria. Similarly, mutant I431V was detected in the absence of K540E. There were eight haplotypes harboring the I431V mutation. The most common haplotypes were **VAG**K**GS** (7.5%, 35/469) and **VAG**KAA (2.1%, 10/469), and were mainly observed in Nigeria. Nevertheless, the effect on SP chemoprevention of the I431V mutation and haplotypes containing it remains unknown.

The quintuple mutant haplotype I_51_R_59_N_108_ - G_437_E_540_ is considered the fully SP-resistant haplotype. This haplotype is reportedly uncommon in West Africa compared with eastern and southern Africa^39^. Similarly, the frequency of I_51_R_59_N_108_ - G_437_E_540_ was just 3.4% in our study and was identified in five countries, occurring most often in Liberian samples. As the mutants *Pfdhps* A581G and A613S were detected in the absence of K540E in West Africa, the super resistant haplotype was not found in our study. The partially resistant haplotype was most prevalent in West Africa (72.6%), with the predominant haplotypes being I_51_R_59_N_108_ - G_437_ (39.1%) and I_51_R_59_N_108_ - A_436_G_437_ (16.0%). The identified haplotype containing the most mutant codons is the octuple mutant haplotype I_51_R_59_N_108_ - V_431_A_436_G_437_G_581_S_613_. It occurred at a frequency of 7.3%, mostly in Nigeria. While the octuple mutant haplotype was limited in the West and Central Africa regions^17^, it may spread to other regions soon. It is urgent to make a surveillance of the prevalence of the octuple mutant haplotype and its effects on SP.

SMC is the intermittent administration of a curative dose of antimalarial medicine during the malaria season, regardless of whether the child is infected with malaria. The objective of SMC is to establish antimalarial drug concentrations in the blood that clear existing infections and prevent new ones during the period of greatest malaria risk. Some prospective trials and ecological studies of SMC with SP + amodiaquine (AQ) in West Africa have reported increased prevalence of the dhfr/dhps quadruple and quintuple mutants, other studies have found no evidence of selection^39^. No evidence has been reported of SMC being followed by increased prevalence of the higher level resistance mutations that most severely impair curative SP efficacy, nor does SMC appear to select for parasites carrying mutations associated with diminished AQ susceptibility. The ability of SP to clear existing infections and prevent new ones is compromised in areas of high to very high resistance, but the intervention still reduces low birthweight and maternal anaemia. So, SP should continue to be used in areas of high SP resistance until more effective alternatives for malaria chemoprevention are found.

This study was limited by some factors. First, the isolates were from Chinese citizens returning from West Africa and not from infants and pregnant women in West Africa. Thus, these samples could be used only to evaluate the molecular epidemiology of SP resistance, not to investigate the efficacy of SP chemoprevention. Furthermore, the samples were not obtained using statistical sampling methods. They were passively collected from returning citizens, and sample sizes were uneven among the countries as well as the years, especially from 2020 to 2022. This might explain why the mutant prevalence in the *Pfdhps* gene, including I431V, S436A/F, A437G, K540E, A581G, and A613S alleles, all showed a trend toward increasing from 2012 to 2022; however, the changes were not statistically significant.

## Conclusion

Our study observed a high mutant prevalence of the *Pfdhfr* and *Pfdhps* genes associated with SP resistance among the *P. falciparum* isolates imported from West Africa during 2012–2022. The mutant prevalence of single nucleotide polymorphisms in the *Pfdhfr* gene had sustained a high level for the 10-year period, while mutants in the *Pfdhps* gene showed an increasing trend. The mutant *Pfdhps* I431V, A581G, and A613S alleles had the highest frequency in Nigeria compared with other countries, while the mutant *Pfdhps* K540E was at the highest frequency in Liberia. The partially resistant haplotype was the most common in West Africa. The fully resistant haplotype I_51_R_59_N_108_ - G_437_E_540_ was at a low prevalence of 3.4% and mainly occurred in Liberia, and the super resistant haplotype was not found in our study. The mutant *Pfdhps* I431V and the octuple mutant haplotype I_51_R_59_N_108_ - V_431_A_436_G_437_G_581_S_613_ showed an increasing trend, and more research about this octuple mutant and its effects is urged. Although the fully resistant haplotype had low prevalence and the super resistant haplotype was rare, the partially resistant haplotype was common. SP should continue to be used in areas of high SP resistance until more effective alternatives for malaria chemoprevention are found. Meanwhile, it is essential to continuously monitor the status of the *Pfdhfr* and *Pfdhps* genes in West Africa with the goal of better implementation of preventive treatment policies for infants and pregnant women.

## Methods

### Sample collection

Data about the imported *P. falciparum* cases were collected from the Disease Surveillance Information Report Management system of the China Center for Disease Control and Prevention. Anticoagulated whole blood samples were collected from patients. The patients were diagnosed with malaria initially by blood smear microscopy and/or rapid diagnosis test (RDT) in the local hospital or County Centers for Disease Control and Prevention. The anticoagulated whole blood and blood smear samples of the cases were collected before antimalarial treatment and deposited in the Sample Resource Library in Henan Provincial Reference Laboratory for Malaria Diagnosis. All of the cases were confirmed to be infected with malaria parasite species using nested PCR (in 2011–2017) or qPCR (after 2017) and blood smear microscopy performed at the Henan Provincial Reference Laboratory for Malaria Diagnosis.

### DNA extraction and amplification

The genomic DNA was extracted from the whole-blood samples using QIAamp DNA Blood Mini Kits (Qiagen Inc., Germany) according to the manufacturer’s instructions. The target genes *Pfdhfr* and *Pfdhps* were amplified using nested PCR with previously described primers and conditions^40,41^. The amplified sequence of the *Pfdhfr* gene contained the codons 16, 51, 59, 108, and 164, and the *Pfdhps* gene harbored the codons 431, 436, 437, 540, 581, and 613. The secondary PCR products were sequenced bidirectionally by Sangon Biotech Co., Ltd. (Shanghai, China).

### Data analysis

The forward and reverse sequences of the *Pfdhfr* and *Pfdhps* genes were assembled through the ChromasPro software version 1.5 (https://technelysium.com.au/wp/chromaspro/). MEGA7 (Molecular Evolutionary Genetics Analysis, https://www.megasoftware.net/show_eua) software was used to identify the mutations by aligning the amplified sequence with the reference genomes. The reference genomes of *Pfdhfr* and *Pfdhps* were retrieved from Genbank, and the Genbank IDs of the *Pfdhfr* and *Pfdhps* genes were PF3D7_0417200 and PF3D7_1324800, respectively. Chi-square or Fisher’s exact test was used to compare differences in prevalence using SPSS version 21.0 (Statistical Product and Service Solutions), and a two-sided *p*-value of < 0.05 was considered statistically significant.

## Data Availability

The original data supporting the conclusions of this article will be made available, further inquiries can be directed to the corresponding authors.
